# 622 Enhancing Confidence and Collaboration Among Burn ICU Nurses

**DOI:** 10.1093/jbcr/iraf019.251

**Published:** 2025-04-01

**Authors:** Taylor Powell, Jennifer Hiner, Maggie Braidwood

**Affiliations:** Regional Burn Center at Harborview; Harborview Medical Center; Harborview Medical Center

## Abstract

**Introduction:**

Current nurse onboarding in the burn intensive care unit (ICU) provides a focused curriculum on critical care, burn resuscitation, and wound care. Opportunities to expand knowledge of the continuation of care after onboarding are limited. This research aims to strengthen collaboration within a burn ICU by adhering to the American Association of Critical-Care Nurses’ six healthy work environment (HWEAT) standards. The project seeks to enhance nurses’ understanding of the multidisciplinary aspects of burn care, improve their knowledge around burn recovery, and foster a collaborative approach essential to successfully caring for burn injured patients.

**Methods:**

The burn ICU’s 2023 HWEAT survey results were analyzed. Categories with a mean score below 3.0 were identified as “need improvement.” Scores between 3.0-3.99 were considered “somewhat healthy”, and scores 4.0-5.0 were “moderately healthy- very healthy.” Additionally, a 5-item Likert scale survey was distributed to all staff to measure their perceived confidence in caring for burn patients and their interest in additional burn care education.

**Results:**

Eighteen respondents participated in the 2023 HWEAT survey. Among the six HWE standards, true collaboration received the lowest mean score (3.93 out of 5.0). The burn ICU scored 4.28 as an aggregate of the HWE standards. Thirty nurses responded to the confidence survey. Opportunities for improvement were noted in the domains of identifying healing progression of a burn injury (3.7) and identifying post operative dressings (3.8).

**Conclusions:**

Burn ICU nurses seek to be less siloed through tighter collaboration and gain better understanding of the other roles within the larger center. Improving confidence in all aspects of burn care and recovery requires an understanding of the multidisciplinary approach that is critical to the management of these patients.

**Applicability of Research to Practice:**

ICU nurses reported a high level of interest in enhancing interdisciplinary collaboration and burn-related education among all ancillary services at the Burn Center. An immersive education pilot is being initiated by the burn ICU to foster collaboration, address knowledge gaps, and offer learning opportunities outside of the ICU setting for our experienced (more than two years) ICU RNs.

**Funding for the Study:**

N/A

